# CdSSe nanowire-chip based wearable sweat sensor

**DOI:** 10.1186/s12951-019-0480-4

**Published:** 2019-03-26

**Authors:** Min Zhang, Shuai Guo, Dieter Weller, Yan Hao, Xianshuang Wang, Chunjie Ding, Ke Chai, Bingsuo Zou, Ruibin Liu

**Affiliations:** 1 Beijing Key Laboratory of Nanophotonics and Ultrafine Optoelectronic Systems, School of Physics, Beijing Institute of Technology, Beijing, 100081 People’s Republic of China; 2 Faculty of Physics and Center for Nanointegration (CENIDE), University of Duisburg-Essen, 47057 Duisburg, Germany

**Keywords:** Wearable sweat sensor, CdSSe nanowire chip, Humidity sensing, Salt sensing, Sweat monitoring

## Abstract

**Background:**

Sweat, as an easily accessible bodily fluid, is enriched with a lot of physiological and health information. A portable and wearable sweat sensor is an important device for an on-body health monitoring. However, there are only few such devices to monitor sweat. Based on the fact that sweat is mainly composed of moisture and salt which is much more abundant than other trace ions in sweat, a new route is proposed to realize wearable sweat sensors using CdSSe nanowire-chips coated with a polyimide (PI) membrane.

**Results:**

Firstly, the composition-graded CdS_1−x_Se_x_ (x = 0–1) nanowire-chip based sensor shows good photo-sensitivity and stress sensitivity which induces linear humidity dependent conductivity. This indicates good moisture response with a maximum responsivity (dI/I) 244% at 80% relative humidity (RH) even in the dark. Furthermore, the linear current decrease with salt increase illustrates the chip sensor has a good salt-sensing ability with the best salt dependent responsivity of 80%, which guarantees the high prediction accuracy in sweat sensing. The sensor current is further proven to nonlinearly correlate to the amount of sweat with excellent stability, reproducibility and recoverability. The wearable sweat sensor is finally applied on-body real-time sweat analysis, showing good consistence with the body status during indoor exercise.

**Conclusions:**

These results suggest that this CdSSe nanowire-chip based PI-coated integrated sensor, combined with inorganic and organic functional layers, provides a simple and reliable method to build up diverse portable and wearable devices for the applications on healthcare and athletic status.

**Electronic supplementary material:**

The online version of this article (10.1186/s12951-019-0480-4) contains supplementary material, which is available to authorized users.

## Background

In recent years, the challenges of sub-health fatigue, aging of raising population as well as prevalence of chronic diseases, need perfect healthcare systems to support person’s fitness. In response to these challenges, flexible and wearable sensors are being paid more attention due to their potential use for healthcare and disease diagnosis [1–4]. Recently, lightweight, ultra-integrated portable, non-invasive devices have been reported in succession [4, 5]. They can be used to monitor various health information of the human body, including glucose, pulse, temperature, blood pressure and blood oxygen [6–12]. These new devices greatly improve the quality of patient care and provide good help for disease diagnosis, treatment and health monitoring, which can improve effective disease and patient management. In the near future, with the development of portable and wearable devices, it can be envisaged that healthcare devices with preventive and precise performance will be utilized in many healthcare fields.

In real life, the most common way to detect health conditions is based on drawn blood, but this invasive method obviously leads to inconvenience, pain, and fear for patients [4]. In fact, sweat, as an easily accessible bodily fluid, is also enriched with a lot of physiological and health information [13–15]. Sweat is a clear, hypotonic, odorless physiological mixture that is commonly considered to be an ultra-filtration of plasma, and it contains important biomarkers, including glucose, lactate, and different types of ions such as Ca^2+^, Na^+^, K^+^, and Cl^−^ [16–19]. Sweat biomonitoring arguably has the greatest potential to evaluate physical conditions in athletes, soldiers, first responders, as well as to detect drug abuse for athletic status optimization [20]. Rapidly growing interest in the physiological information including sweat has gradually led to sensor promotion for secretion [21]. As is well known, the most abundant and important ions in sweat are Na^+^ and Cl^−^, which compose salt. They manage the production and secretion of sweat. When the sweat rate increases, Na^+^ and Cl^−^ increase in the final secreted sweat [22].

For real medical convenience, a flexible wearable sensor, with patch-style formats to realize real-time sweat monitoring, is preferable and can discreetly adhere to the skin without much influence by users’ activities [21]. This could make an on-body sweat sensor provide real-time and continuous information to continuously evaluate person’s fitness, including the different stages of dehydration (hypertonic, hypotonic, and isotonic dehydration) that can induce performance loss, nausea, headaches and even death [18]. Currently, commercially wearable sensors to monitor heart rate and blood pressure are popularly utilized [17, 23]. Wearable sweat sensors are still relatively few, even though their methods and mechanisms are very relevant. They include functionalized electrochemical detection sensors to transduce analytic and colorimetric detection by analyzing related color changes of target reagents [21, 24], impedance-based and optical sensing [25–27], as well as use of microfluidics and multiplexed sensing [21, 28]. The common and versatile methods discussed above show great utility to detect specially appointed ion species in sweat that mainly depend on the electrochemical sensor. Thus, it is important to develop a wearable sweat sensor for reliable sweat monitoring, especially for direct monitoring of the sweat rate.

CdSSe nanostructures are often used to study their good optoelectronic properties due to their excellent transport properties and tunable bandgap [29–32]. Here, a fresh new idea and easy way is utilized to realize wearable devices by an inorganic and organic composite. We successfully fabricated an in situ portable and wearable sweat sensor by using ternary CdS_1−x_Se_x_ (x = 0–1) nanowire alloy chips coated with a polyimide (PI) layer thereby finishing the moisture and salt sensing with performing further sweat monitoring. The CdSSe nanowire chip-based multilayer sensor combines by the organic polymer and inorganic semiconductor layer by nanowire integration with mica as substrate. The nanowire chip with good flexibility is utilized to realize the fabrication of a wearable sweat sensor. The moisture sensitive material, PI layer coated on the surface of the CdSSe nanowire chip, generates hygroscopic expansion to induce stress on the attached CdSSe nanowire layer. This causes the conductivity to change as the moisture dependent current of the sensor increases with rising humidity. The linear dependence of the moisture on the resistance makes the humidity prediction very precise. Furthermore, the conductivity of the PI coated CdSSe nanowire-chip is also sensitive to the deposited salt on the surface. The experiment by dropping sweat directly onto the surface of the sensor proves sensitive dependence of sensor current on the amount of sweat, i.e. a mixture of water and salt. Finally, a real-time on-body sweat monitoring is used to reveal the perspiration status of the tester. According to the measured results, the human body’s sport status at different stages is predicted and summarized. Therefore, depending on the good response on moisture and salt, a cheap and easy PI coated CdSSe nanowire chip is successfully utilized for sweat sensing for healthcare.

## Results and discussion

### PL and SEM analysis

**Fig. 1:**
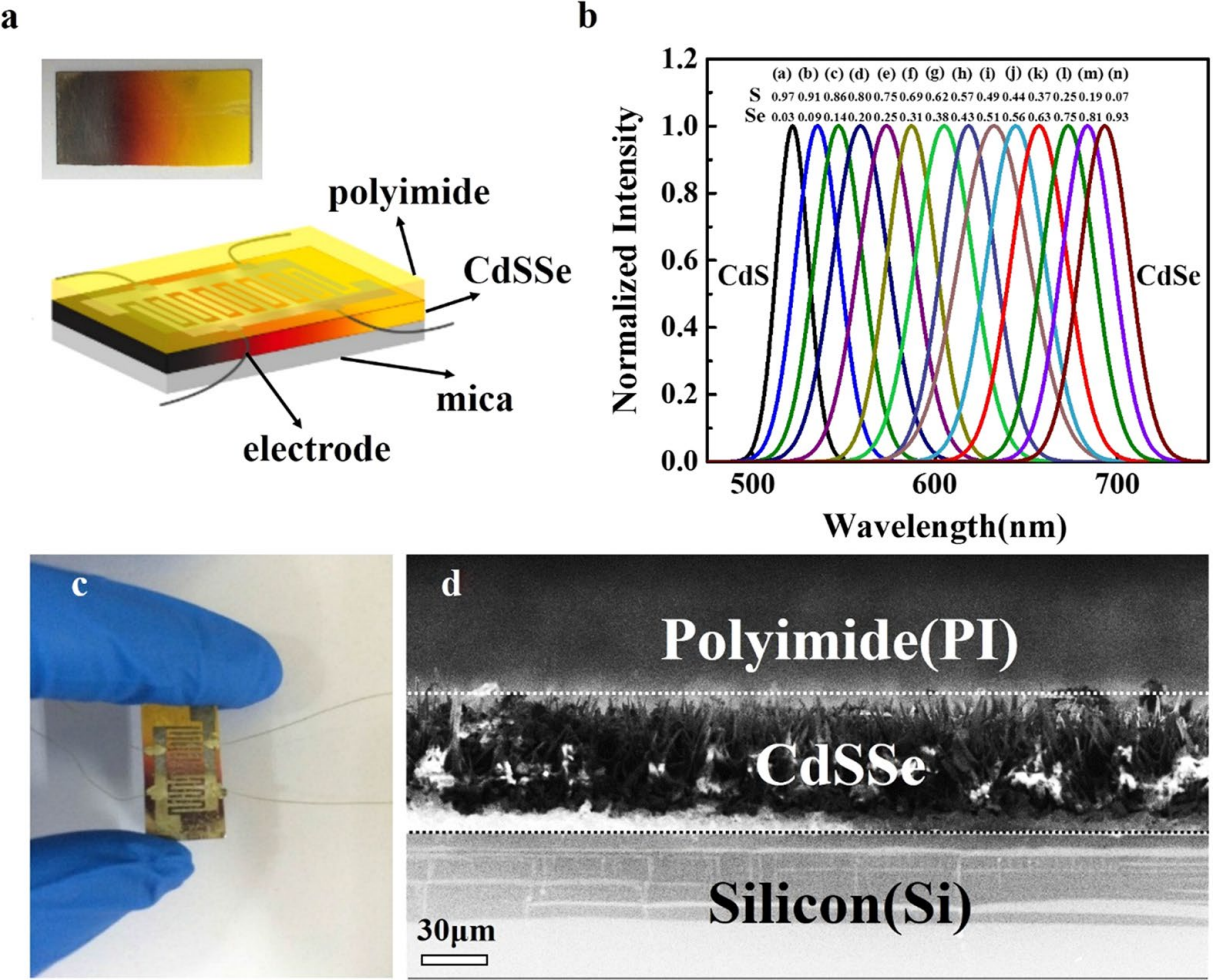
**a** Real-color photograph of tunable-composition ternary alloy CdS_1−x_Se_x_ chip and the diagram of the sweat sensor. **b** PL spectra measured at different composition along the length direction of the CdSSe nanowire chip by a laser at 405 nm. **c** Real image of the as-prepared sweat sensor. **d** Cross-sectional SEM image of silicon (Si) substrate based PI-coated CdSSe nanowire chip. Scale bar: 30 μm

The CdSSe nanowire chip with the size of 1 cm × 2 cm obtained by the CVD method is shown in Fig. 1a, and the color of the as-prepared sample changes gradually from black to yellow along the length direction of the chip. It proves that the composition of the nanostructures changes gradually. Figure 1a also shows the structural diagram of the CdSSe nanowire chip-based sensor. Three layers are included, upper PI layer, middle CdSSe nanowire part, and soft mica substrate, among them, the functional layer for moisture monitoring is PI that can induce stress on the conductive nanowire layer due to the moisture induced expansion in the PI layer, causing the conductivity change, thus to realize moisture sensing. Figure 1b shows the normalized PL spectra from the CdSSe nanowire chip under the irradiation of a 405 nm laser line. The peaks gradually change from 512 nm of CdS to 700 nm of CdS_0.07_Se_0.93_, which proves that the alloy composition as well as the band gaps is gradient along the long side of the rectangular substrate. Figure 1c shows the real image of the as-prepared sensor based on the CdSSe nanowire chip. A key requirement for wearable sensors is the flexibility without any influence with user movement, which should release the mechanical strains and distortions with on-body use. Therefore, the mica is proper as the substrate to make the device with a good flexibility and proper bearing performance as wearable device. The CdSSe nanowire chip demonstrates a better conductivity after applying the external stress, which makes the nanowire with different composition contact to each other quite well (Additional file 1: Figure S2). The cross-section of the nanowire-chip based sensor, with Si rather than mica as the substrate in Fig. 1d for the conductivity consideration in SEM measurement, illustrates that the CdSSe nanowires and PI fully contact together, which guarantees the stress generated from PI can well interact onto the CdSSe nanowires. More importantly, the PI coating should be uniform to ensure that the stress applied on the CdSSe nanowires homogeneously. The full contact of each layer also avoids delamination.

### Humidity sensing

**Fig. 2:**
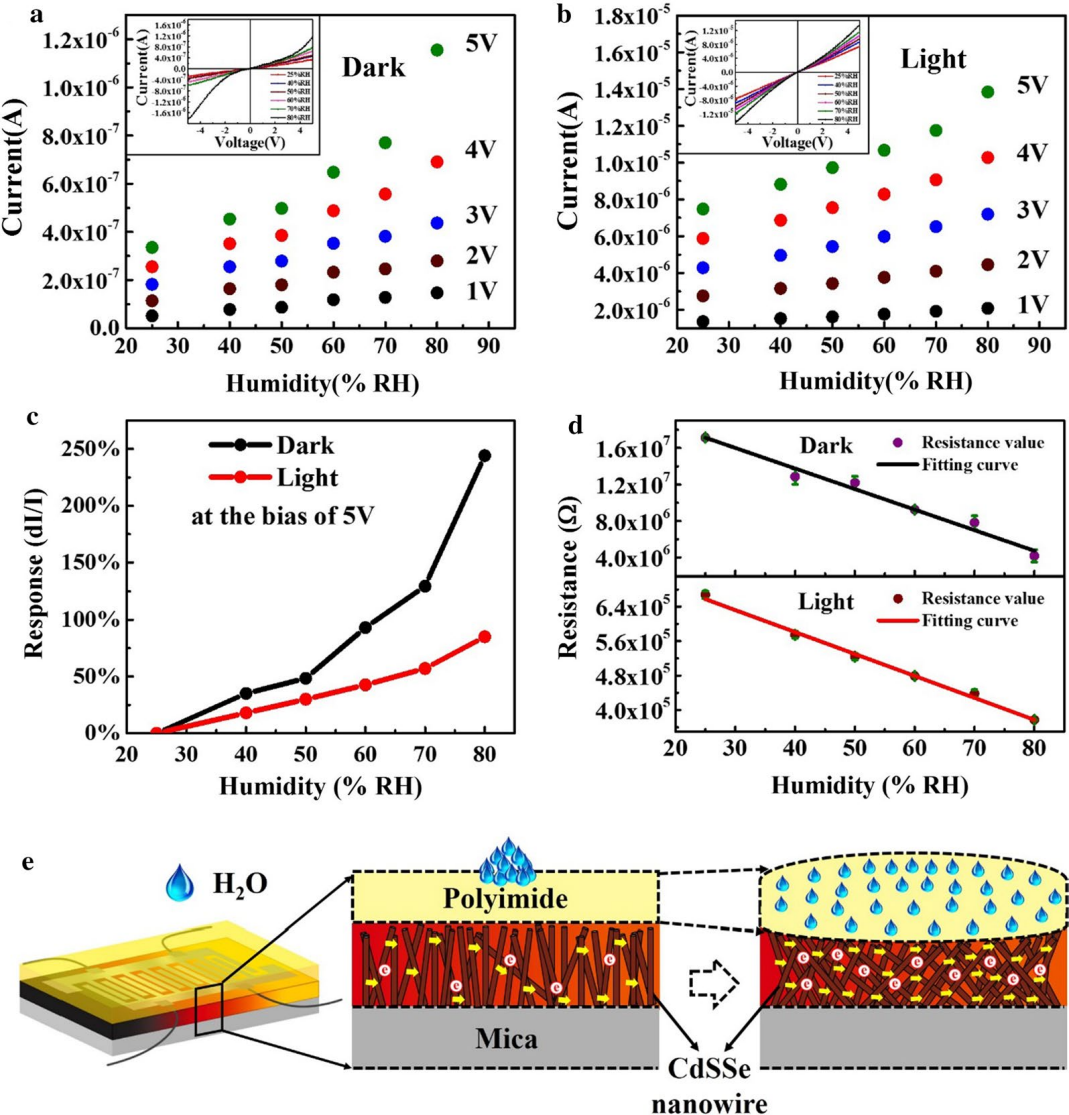
Dark current (**a**) and photo-current (**b**) at various humidity levels under different bias. The insets: corresponding I–V curves of the sensor in the dark (**a**) and light (**b**) surrounded by different relative humidity. **c** Relative humidity dependent moisture responsivity of the sensor in the dark and bright fields, respectively. **d** Resistance at various relative humidity of the sensor in the dark and bright fields, respectively. **e** The schematically theoretical diagram of humidity sensing of the sensor

Sweat evaporation is a slow and gradual process to the skin surface, with the volume increase of sweat moisture, the sweat bursts through the skin and forms sweat beads between the pores and the surface of the skin. Therefore, the CdSSe chip sensor should firstly respond to the moisture. Then the response characteristics to the moisture were simulated in a sealed chamber in order to eliminate the influence of ambient humidity and temperature, as shown in Fig. 2, it proves that the sensors have good and fast response to the sweat-like moisture. As shown in Fig. 2a, b, the humidity dependent current at various bias from 1 to 5 V is demonstrated in both dark and light conditions, respectively. The response current increases in a monotonic manner with the RH of moisture raising under each specific bias. At 5 V in the dark, the current value sharply increases from 3.36 × 10^−7^ to 1.15 × 10^−6^ A with the RH increasing from 25 to 80%, as shown in Fig. 2a, and upon illumination, the sensor has a better linear dependence with the photocurrent changing from 7.48 × 10^−6^ to 1.38 × 10^−5^ A under 5 V bias, as shown in Fig. 2b. The insets of Fig. 2a, b show the measured current–voltage (I–V) curves of the device under various moisture levels in the dark and bright fields, respectively. The sub-linear dependence on the increasing moisture of the device demonstrates that the sensor can work day and night with similar response performance. Figure 2c shows the RH dependent responsivity of the sensor under 5 V bias. It is defined as [(I_various RH_ − I_RH=25%_)/I_RH=25%_], where I_various RH_ is the current at various RH, and I_RH=25%_ is the current at 25% RH. The maximum responsivity of current change being 244% in the dark at 80% RH can be calculated, which illustrates the CdSSe nanowire chip has an ultrasensitive response on the moisture variation under proper bias. The reason is that the PI coating layer can adsorb water molecules of surrounding moisture and then the volume expands to generate mechanical stress on the CdSSe nanowire layer [33, 34], as shown in Fig. 2e. The pressure at the interface between the PI and CdSSe layers further tightens the contact between the nanowires, thus improving the interconnection conditions and shortening the transportation paths. It also makes the contact barriers narrower and lower to greatly enhance the current. The larger swelling level of PI generates more pressure force on the CdSSe nanowires. Accordingly, the current of the device increases with raising humidity. Under the same RH, the dark current is one order of magnitude smaller than that of the photocurrent in the bright field for the contribution of photo-induced carriers.

Figure 2d shows the resistance of the sensor under different relative humidity levels. The resistance of the device monotonously decreases from 1.71 × 10^7^ to 4.18 × 10^6^ Ω in the dark field and from 6.68 × 10^5^ to 3.77 × 10^5^ Ω in the bright field, respectively, as the relative humidity increases from 25 to 80%. The linear dependence of resistance on the moisture RH of the CdSSe nanowire chip sensor for both darkness and lightness is feasible and convenient to be used as standard calibration curve to predict the humidity of moisture. The RH of moisture dependent resistance in the dark field can be well fitted with a linear function with the goodness of the fit R^2^ of 0.9779. Upon illumination, a better linear fitting of the resistance-humidity curve has been established with R^2^ of 0.9948. It is noteworthy that our sensors have a higher linear relationship compared to the other reported humidity sensors [35, 36], which means better prediction ability on quantitative analysis of the sweat moisture. In addition, after repeated tests, the device works well with excellent reproducibility of humidity sensing and different devices have the similar response characteristics, as shown in Additional file 1: Figure S3. Repeated experiments show that the sensor’s perceived minimum humidity level is about 20% RH due to insufficient polyimide moisture absorption, which causes rare changes in the sensor conductivity. The low humidity sensing limit of the sensor makes it possible to detect tiny amounts of sweat evaporation in the human body.

### Salt sensing

**Fig. 3:**
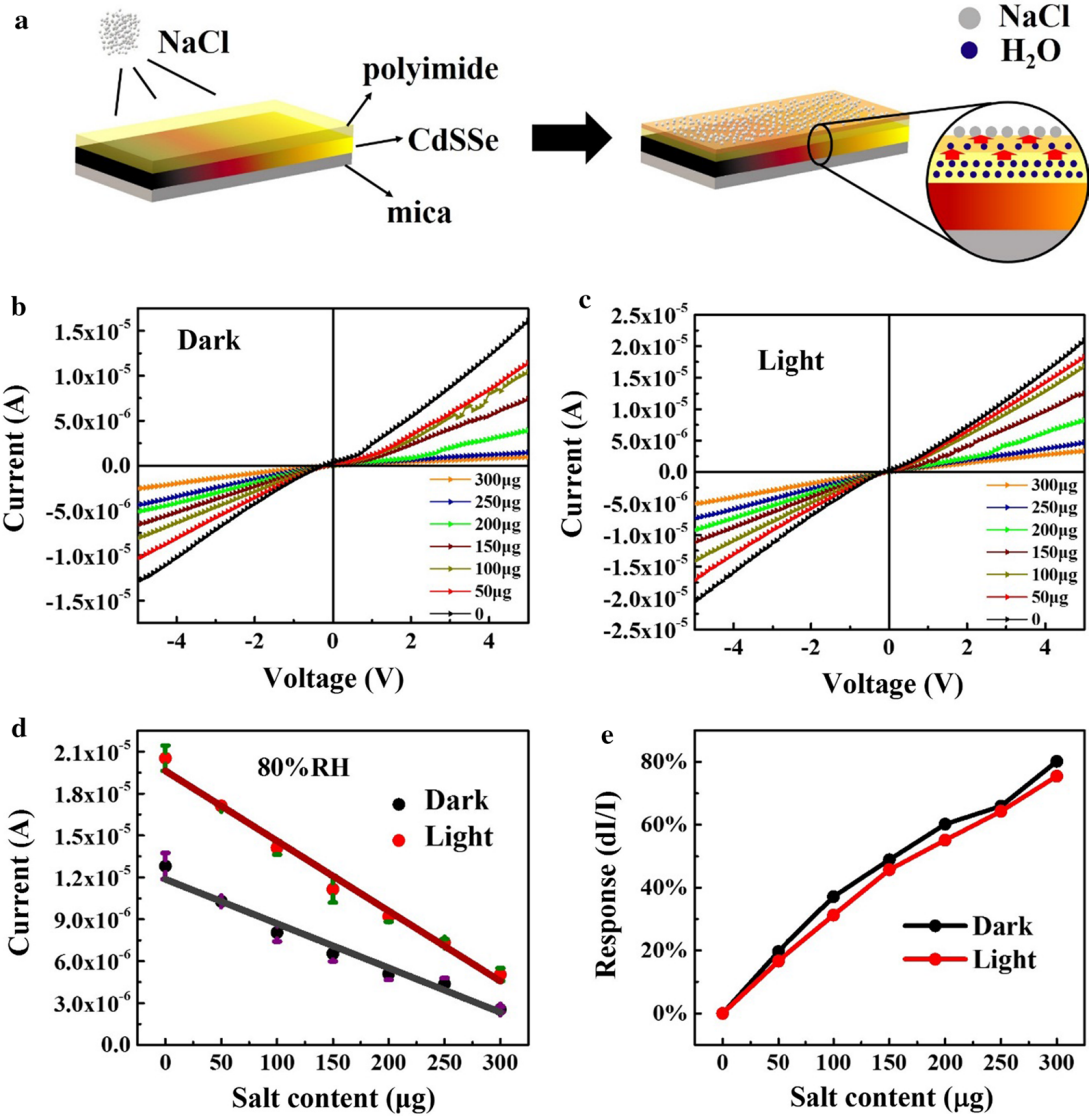
**a** Salt-sensing process and mechanism of a CdSSe-based sensor. I–V characteristic curves of salt sensing in the dark (**b**) and light (**c**) under different salt content levels at high humidity of 80% RH. **d** The current variation of the sensor with the salt content at a bias of 5 V. **e** The salt content dependent responsivity of the CdSSe-based sensor

In addition to the moisture response, the CdSSe nanowire-based sensors also respond well to soluble salt (NaCl) in sweat. The schematic diagram of salt-sensing process and mechanism of the CdSSe-based sensor is shown in Fig. 3a. The layered structure is still composed by the transparent PI membrane on top, CdSSe nanowire chip in the middle, and soft mica as substrate. Figure 3b, c show the I–V characteristic curves of the sensor under different NaCl content levels at a fixed high humidity of 80% RH in the dark and light, respectively. At 5 V bias, the current reduces linearly with the increase of salt content in both dark and light as shown in Fig. 3d. The trend of the I-V curves is opposite to the humidity dependent current (see in Fig. 2). Figure 3e shows the corresponding responsivity of the salt sensing at the bias of 5 V, which is defined as [(I_salt content=0 μg_ − I_various salt content_)/I_salt content=0 μg_]. With the salt content increase, the response of the salt sensing linearly increases in both dark and light conditions. The maximum responsivity reaches 80% and the change rate is as low as 0.3%/μg, which illustrates the prepared sensors are sensitive to the salt. It is because the salt has an impact on the hygroscopic swelling effect of the PI film, that is, the expansion of PI induced by water adsorption can be restrained by salt, as the schematic diagram shown in Fig. 3a, where the sensor is placed in an environment with fixed humidity and the water molecules rapidly diffuse into the PI film to induce the expansion of the film. After the current becomes relatively steady when the surface of the PI film is sprayed with NaCl particles, the outer part of the film forms a NaCl solution by adsorbing the moisture from PI and surrounding moisture. This is expressed by the deep color part of the PI film indicating the concentration difference between in- and outside the film. Accordingly, the water inside the film will gradually spread to the outer layer until it is in balance between the inner and outer layer. In this case, the degree of expansion of the inner film is correspondingly reduced so that the stress applied on the CdSSe nanostructures also decreases accordingly. As is known to all, PI has a moisture-sensitive center, carboxyl group (–COOH), which determines the water adsorption capacity by the affinity. The more carboxyl groups (–COOH) are included, the better is the moisture sensitivity. As a kind of strong polar molecule, carboxyl groups (–COOH) combine with water molecules by the van der Waals force, and meanwhile, the water molecules connect each other by hydrogen bonds interaction to form clusters to induce continuous adsorption on the surface of the PI membrane. However, the Na^+^ and Cl^−^ in NaCl solution have been proved to damage the hydrogen bonds in H_2_O and that will be enhanced with increase of Na^+^ and Cl^−^ concentration [37–39]. Thus, in high RH, the PI membrane of the sensor absorbs water and swells, while NaCl can destroy the formation of hydrogen bonds and therefore limits the water adsorption by the carboxyl groups (–COOH), which results in the reduction of the PI expansion rate and the nanowire contact on the chip becomes worse, then making the decrease of the humidity-sensitive responsivity. The reproducibility of this phenomenon is confirmed by repeating experiments with many times, similar results occur, as shown in Additional file 1: Figure S4. The recovery process of the sensors after salt sensing can be found in Additional file 1: Figure S5, and excellent recovery performance contributes to good repeatability. On the basis of the up-mentioned theory, it demonstrates that the sensors can work well for the salt sensing with good repeatability and reproducibility.

### Sweat detection and stability of the sensor

**Fig. 4:**
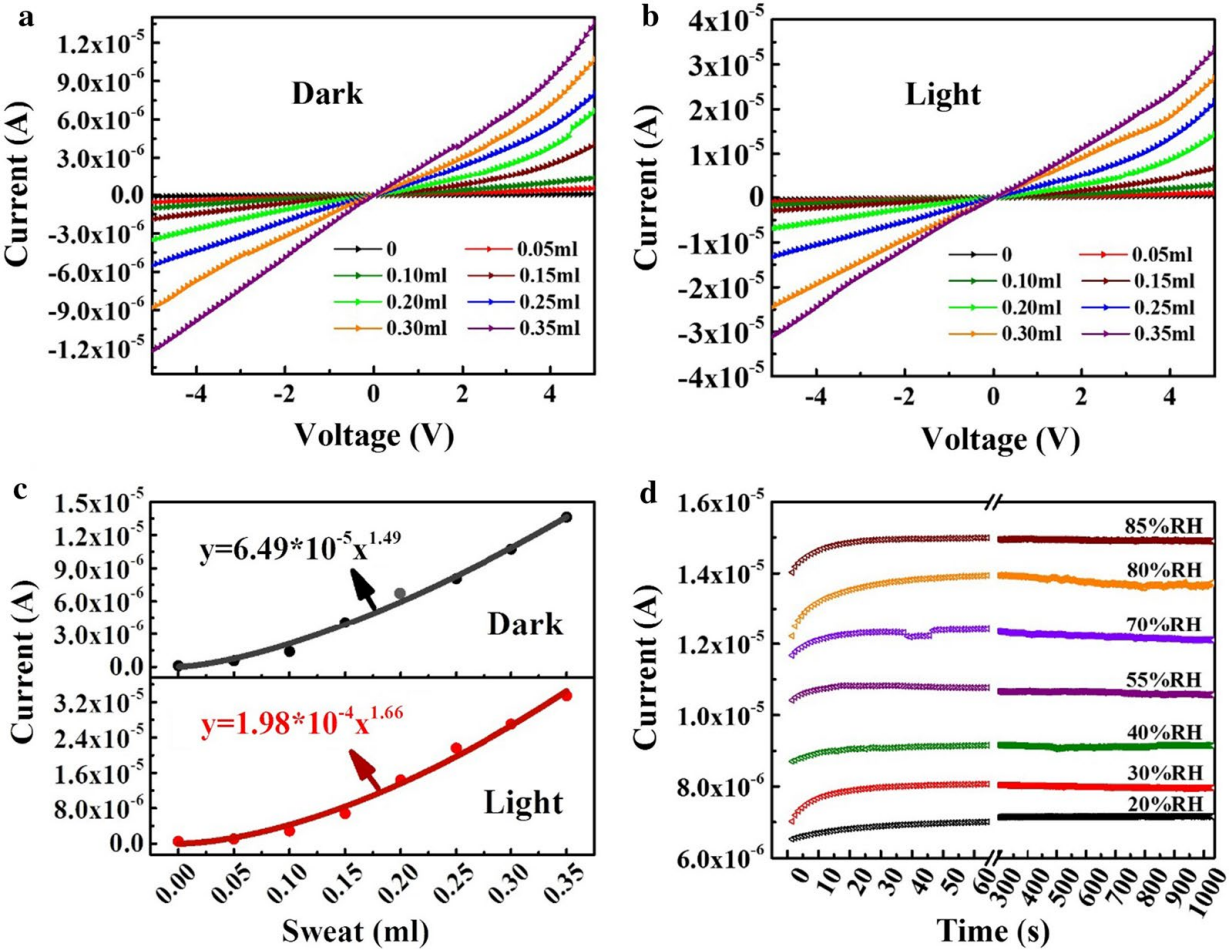
I-V curves of the sensor under different amounts of human sweat in the dark (**a**) and bright (**b**) fields, respectively. **c** Conductive currents under dark and light condition are shown to directly correlate with sweat volume, underscoring the possibility of quantitative analysis of sweat monitoring. The corresponding regression curve was given to predict the volume of sweat by the current of the sensor. **d** Long-term stability of the CdSSe nanowire-chip sensor

By dropping sweat directly onto the surface of the sensor, the dependence of sensor current on the amount of sweat is studied, as shown in Fig. 4. The results of Fig. 4a, b show the I–V curves of the sensor corresponding to various amounts of human sweat in the dark and bright fields, respectively. The sensor current gradually increases with the increase of the amount of sweat, which is due to the increase of the sweat volume and it causes an expansion of the PI layer at various degrees, hence the sensor current increases according to the above rule. Figure 4c indicates that, at 5 V bias, the sensor current increases exponentially with increasing sweat volume. The tendency of both currents changing can be well fitted by an exponential function as dark and red curve described in Fig. 4c, respectively. The similar sensing tendencies on the sweat in the dark and bright fields prove that the sensor can work well day and night. The nonlinear relationship due to the PI expansion is determined by both liquid and salt together in the sweat drops and the nonlinear expansion occurs. As mentioned above in Fig. 3, the water adsorption of PI is a process in which water gradually diffuses into the PI layer through the interaction of intermolecular forces and hydrogen bonds. The larger volume of sweat makes wider effective areas diffused and occupied onto the chip. Therefore, the current increases exponentially with the amount of sweat increasing. These results demonstrate that sweat monitoring and healthcare predictions day and night can be performed by the sensor with a proper calibration curve.

The stability of the sensors is also checked by the time dependent current variations in 1050 s (17.5 min) under different humidity levels of 20% to 85% RH, as shown in Fig. 4d, just considering the impact of moisture on the sensor because the moisture is a main factor to induce the sensing signal change. With the moisture rising, the current of the device gradually lifts to a higher level. For a specific RH, the current variation is less than 3% during the measurement period of 1050 s, which demonstrates that the sensors possess excellent stability during personally physical exercise.

### Real-time on-body sweat monitoring

**Fig. 5:**
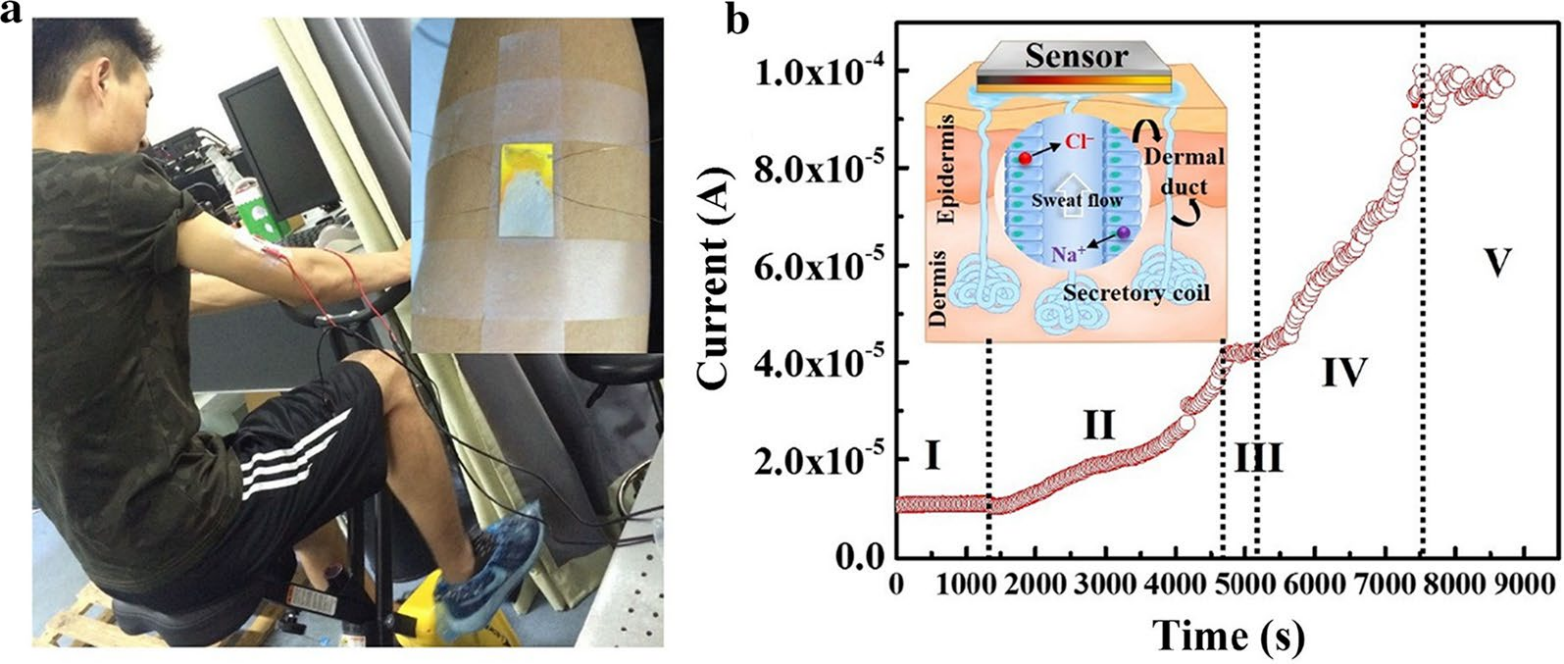
**a** Photograph for real-time on-body sweat monitoring during indoor cycling. The inset: real-time monitoring scene of the sensor attaching to the arm. **b** The data of real-time skin sweat measurement. The inset: schematic diagram of sweating monitoring for sensor attaching to the skin

For continuous fitness monitoring, an actual application of the sweat monitoring is performed by the sensor, by attaching a chip on the arm when a person rides a bicycle to exercise in a closed laboratory where the air conditioner is shut down to enable secrete sweat, as shown in Fig. 5a. The most abundant ion species are Na^+^ and Cl^−^ that effectively guarantee the salt monitoring. The real-time current from the sensor is obtained by on-body sweat monitoring over a period of indoor cycling by using the sweat sensor, as shown in Fig. 5b. The inset in Fig. 5b is the schematic diagram of a sweat monitoring sensor attached to the skin. The relation between sweat analyte levels and health status is still poorly understood. To understand how sweat analytes correlate with blood or IF (intracellular fluid) levels, and hence the utility of probing sweat for medical or fitness monitoring, it is crucial to first understand the mechanisms by which analytes are partitioned into sweat. The perspiration starts after about 20 min of exercise during a constant load as preheating stage I. In this stage, there is no obvious current change being detected from the sensor. After that, a clear increase of sensor current is observed as long as sweat secretes (stage II), which indicates the beginning of the initial sweat. After having a rest for five minutes and 200 ml water intake, a high-intensity exercise with an increased load was carried out by the person. Another rapid increase in sensor current is observed when the person goes through excessive sweating (stage IV), which indicates dehydration of the person. During the resting process, there is almost no current change being observed from the sensor monitoring (stage III). The phenomenon is an important sign to let the person have a rest and drink. About 2 h later, the current tends to be stable (stage V), which demonstrates that the device becomes saturated. Due to Na^+^ and Cl^−^ in the final secreted sweat to typically increase with sweat rate [22], it encourages the chip sensor to be used to monitor the sweat rate. In the future, the sweat sensors as hardware, together with proper software and algorithms, will be functional as wearable electronic devices for the analysis of human sweating conditions to determine whether the body is dehydrated or there is a lack of electrolyte and most importantly to prevent the occurrence of danger. Due to sweat rates varying with activity intensity and hydration level they broadly differ between individuals. For precise quantitative predictions, sensors, that are robust under these conditions as well as the base- and correlation line between sweat content and sensor parameters, need to be set firstly and be enabled to quantitatively detect analytes.

Generally, for on-body sensors, care must be taken to prevent delamination of the flexible components during exercise. For our device, because of the flexible substrate-based sensing components and reasonable structural design, it is ensured that the functional membrane layers deform along with the mica substrate without rupturing and also that the sensor working area does not change dramatically during deformation to avoid motion-related signal artefacts (see detail in Additional file 1: Figures S6 and S7). This is ideal for fitness monitoring, providing profiles of changing analyte concentrations that can inform the user of depleting electrolytes or dehydration. Therefore, our fabricated polyimide coated CdSSe wearable sensor shows good responsivity on sweat compared with other reported wearable sensors (see detail in Additional file 1: Table S1).

## Conclusions

In this paper, a simple large-size moisture and salt sensor for wearable sweat monitoring was designed and fabricated layer by layer. The composition-graded semiconductor CdSSe alloy chips were grown, combining with the polymer hygroscopic material polyimide (PI) as top layer, to successfully prepare sweat sensors. The CdS_1−x_Se_x_ chip is integrated with graded-composition nanowires with the bandgap changing from 1.78 eV of CdSe to 2.42 eV of CdS. The achieved sensors demonstrate good performance of moisture monitoring and sensitive salt responses with stability and reproducibility. The output current signal increases in a monotonous manner when rising the relative humidity, and the largest responsivity is 244% which is acquired at 80% RH in dark. The goodness of fit R^2^ by linear line for the resistance-humidity curve of the sensor is 0.9779 in the dark. Upon illumination, a better linear R^2^ fitting is equal to 0.9948. The effect of salt on the polyimide after hygroscopic expansion is analyzed with the sensor current linearly decreasing as the salt increases, making it a salt-sensor with good sensitivity. Finally, the nonlinear relationship of the amount of sweat, as mixture of water and salt, and the sensor current is found. Based on the good response of the sensor on sweat, an actual sweat sensor works well on real-time sweat on-body monitoring during indoor exercise by attaching the chip on a person’s arm. The proposed sweat sensor shows a promising application in portable and wearable sensor devices. This paper provides a new method to build up a new type of a wearable sensor for healthcare by inorganic and organic composite structures. The potential applications represent some of the opportunities for sweat sensors in the future. These complex correlation studies remain a key challenge for establishing the wider utility of sweat sensing for human health.

## Methods

### Sample growth

The composition-graded CdS_1−x_Se_x_ nanowire chips in this research were synthesized by a chemical vapor deposition (CVD) method using Au as catalyst. A detailed description of the reaction system was given in a previous paper [40]. The mixture of CdS powder (Alfa Aesar, 99.995%) and CdSe powder (Alfa Aesar, 99.995%) with the molar ratio of 1:1 was placed in a ceramic boat, which was in the center of tube furnace. Soft mica wafers coated with an Au catalytic layer as growth substrates, were placed about 10 cm away from the source powders in both sides of the quartz tube. Prior to a rapid heating to 1000 °C within 10 min and being kept at this temperature for 120 min with maintaining the Ar (90%)/H_2_ (10%) flow at 20 sccm, the quartz tube was purged with high-purity Ar (90%)/H_2_ (10%) at a constant flow rate of 60 sccm for 30 min to eliminate O_2_. After the furnace cooled down to room temperature spontaneously, the uniform CdSSe nanostructures with gradient composition were observed to deposit on the substrates.

### Fabrication of sensor

After synthesis of a CdSSe nanowire chip, interdigital Al contact electrodes were deposited on both sides of the nanowire chip by a vacuum thermal evaporation system, then Ag wires were attached to the interdigital electrodes using silver colloid to connect with external equipment. Finally, the polyimide (PI) as moisture sensitive layer, was uniformly coated by a spin-coating method on the surface of an integrated CdSSe nanowire chip to successfully fabricate a complete sweat sensor. The detailed preparation process of the device is shown in Additional file 1: Figure S1.

### Setup of measurement

PL spectra of the CdSSe nanowire chip were obtained using a 405 nm laser as an excitation source. The layered structure of the sensor was characterized by scanning electron microscopy (SEM), and the photoelectric characteristics were measured by a semiconductor test instrument (Keithley-4200).

## Additional file

**Additional file 1: Figure S1.** A schematic illustration of the fabrication process of the device. **Figure S2.** (a): The I-V curves of the sensor under different mechanical pressure in the dark. (b): The dependence of the current on the mechanical pressure at 5 V bias in the dark field. **Figure S3.** The I-V curves from repeated humidity sensing in the dark and in the light, respectively. **Figure S4.** (a): The I-V characteristic curves of the sensor at different concentrations of salt solution under the light. (b): The photocurrent variation under different concentrations at the bias of 5 V. **Figure S5.** (a): The recovery process of the sensor after detecting salt solution at the concentration of 30 g/L. (b): The recovery current of the different concentrations of salt solution. **Figure S6.** The wearable sensor with different bending degree. **Figure S7.** The I-V curves of the sensor with different bending degree at different relative humidity. **Table S1.** Comparison of performance of various wearable sensing mast.
