# Supplementary material for: CdSSe nanowire-chip based wearable sweat sensor
Source: J Nanobiotechnology. 2019 Mar 26;17:42. doi: 10.1186/s12951-019-0480-4 (PMC6434865; doi:10.1186/s12951-019-0480-4)
Supplement: Supplementary file 1 — Additional file 1: Figure S1. A schematic illustration of the fabrication process of the device. Figure S2. (a): The I-V curves of the sensor under different mechanical pressure in the dark. (b): The dependence of the current on the mechanical pressure at 5 V bias in the dark field. Figure S3. The I-V curves from repeated humidity sensing in the dark and in the light, respectively. Figure S4. (a): The I-V characteristic curves of the sensor at different concentrations of salt solution under the light. (b): The photocurrent variation under different concentrations at the bias of 5 V. Figure S5. (a): The recovery process of the sensor after detecting salt solution at the concentration of 30 g/L. (b): The recovery current of the different concentrations of salt solution. Figure S6. The wearable sensor with different bending degree. Figure S7. The I-V curves of the sensor with different bending degree at different relative humidity. Table S1. Comparison of performance of various wearable sensing mast. [file 12951_2019_480_MOESM1_ESM.docx]

**Additional figures for**

**“CdSSe Nanowire-Chip Based Wearable Sweat Sensor”**

Min Zhang^1^, Shuai Guo^1^, Dieter Weller^2^, Yan Hao^1^, Xianshuang Wang^1^, Chunjie Ding^1^, Ke Chai^1^, Bingsuo Zou^1^ and Ruibin Liu^1^*

^1^Beijing Key Laboratory of Nanophotonics and Ultrafine Optoelectronic Systems, School of Physics, Beijing Institute of Technology, 100081, P. R. China

^2^Faculty of Physics and Center for Nanointegration (CENIDE), University of Duisburg-Essen, 47057 Duisburg, Germany

*Corresponding author. Email address: [liuruibin8@gmail.com](mailto:liuruibin8@gmail.com) (R.B. Liu)


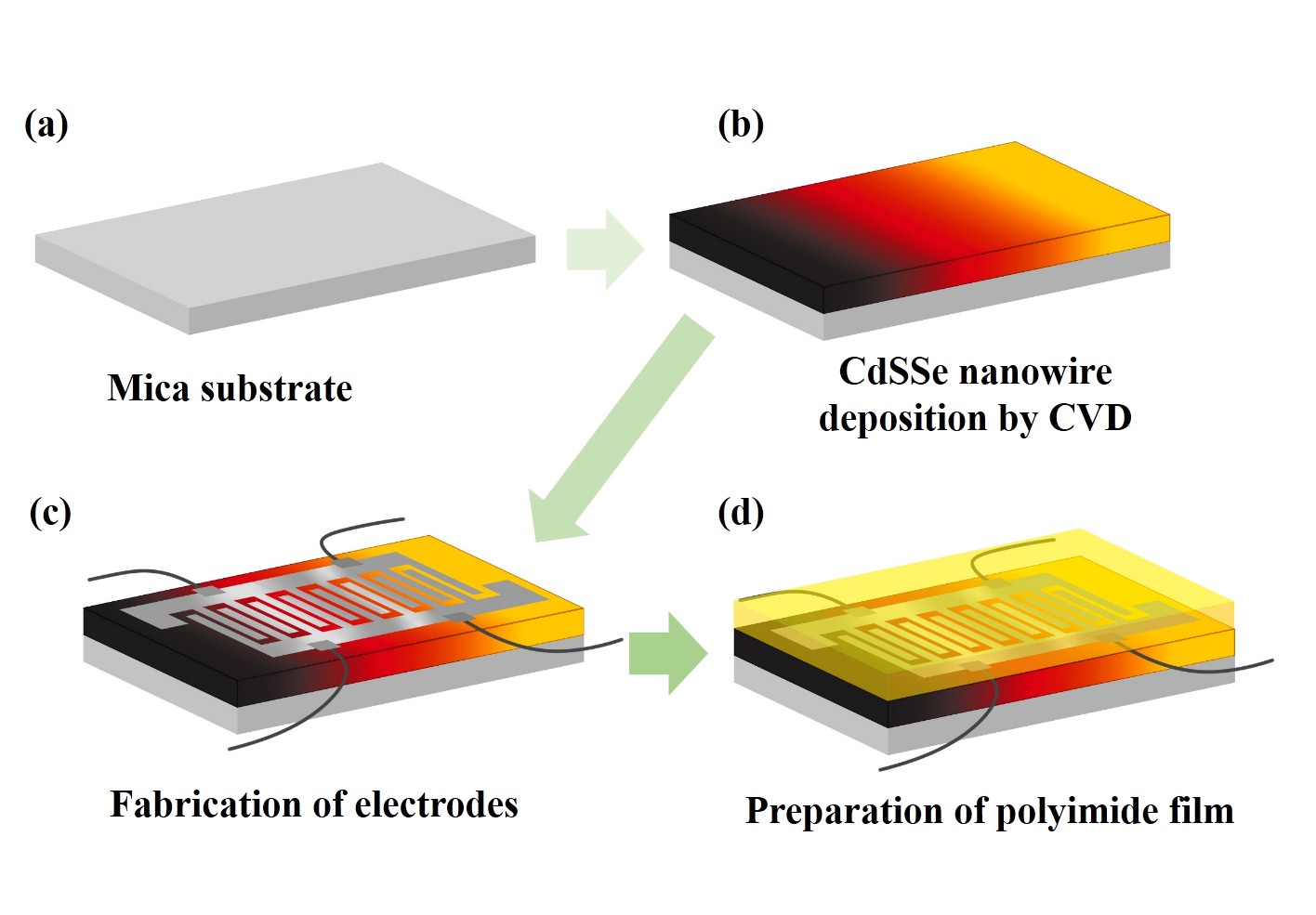


Figure S1 A schematic illustration of the fabrication process of the device.


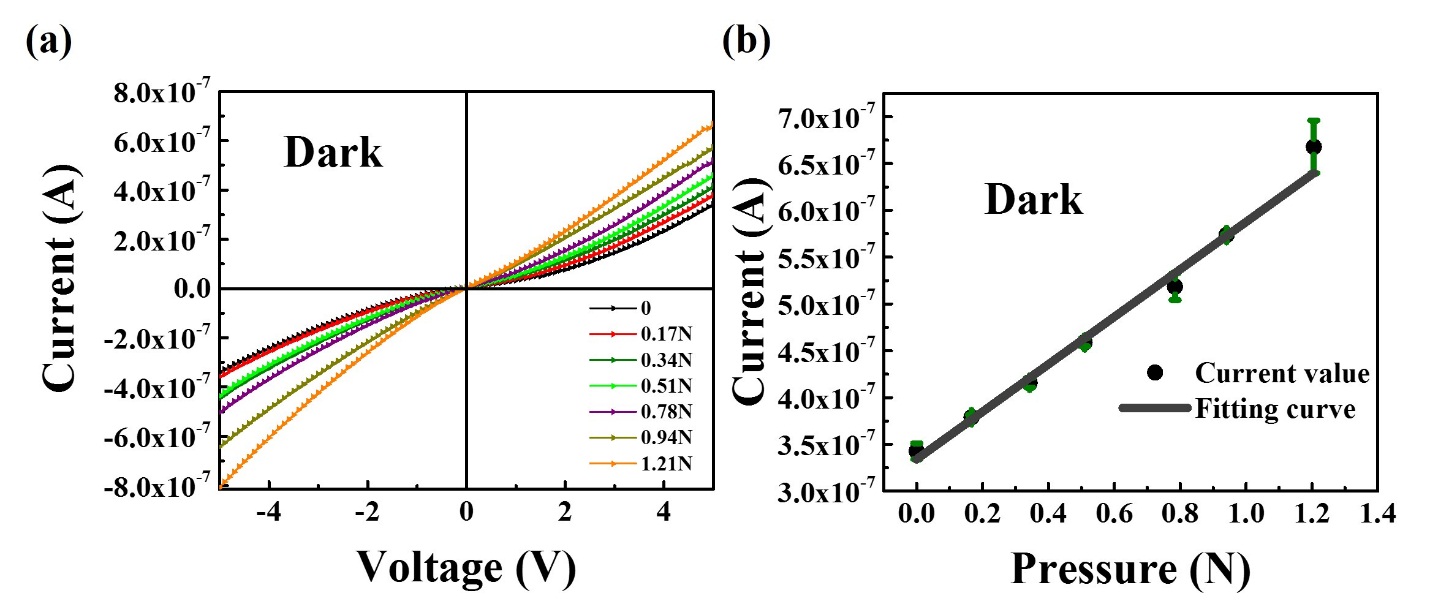


Figure S2 (a) The I-V curves of the sensor under different mechanical pressure in the dark. (b) The dependence of the current on the mechanical pressure at 5V bias in the dark field.

Figure S2 (a) shows the typical current-voltage (I-V) curves under different mechanical pressure applied on the sensor. Figure S2 (b) shows the dependence of the current on the mechanical pressure at 5V bias. It can be seen that the current increases linearly as the pressure increased in Figure S2 (b), which indicates that the external pressure applied on the CdSSe-chip based sensor can induce the linear current change with pressure increase. It is consistent with the current tension variation from PI expansion induced by the humidity.


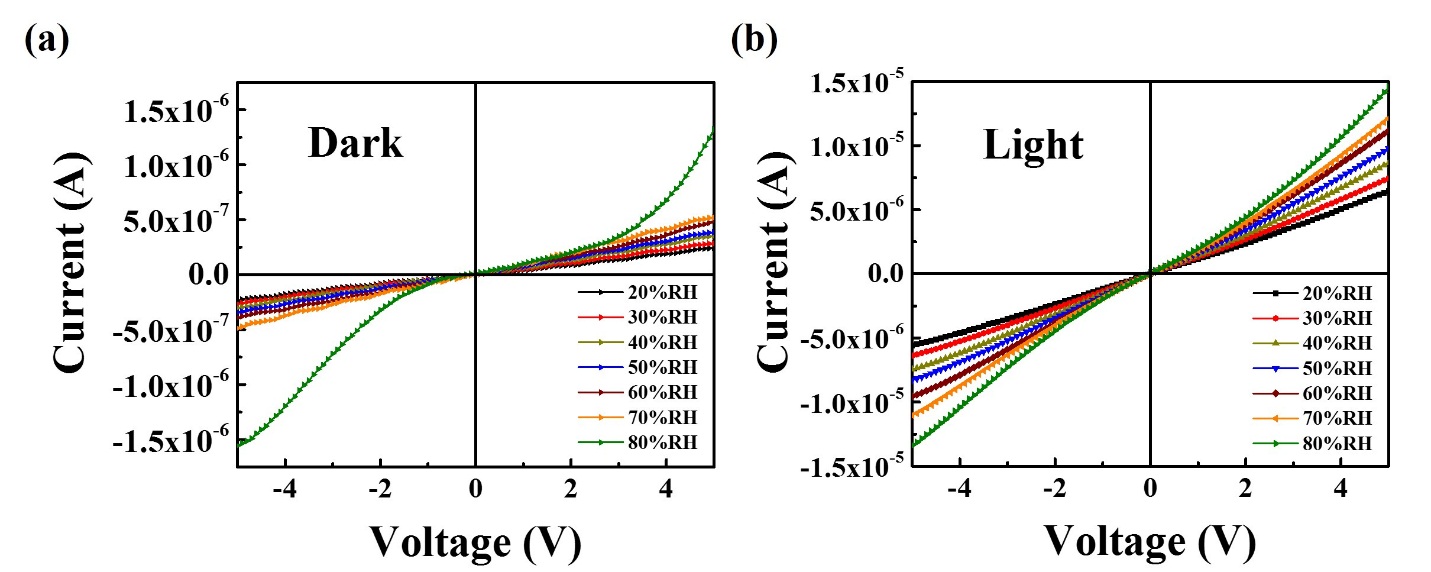


Figure S3 (a) The I-V curves from repeated humidity sensing in the dark and (b) in the light, respectively.

Figure S3 shows the results of humidity detection by another device. After repeated tests, it proves that the humidity sensor is reproducible with good consistency.


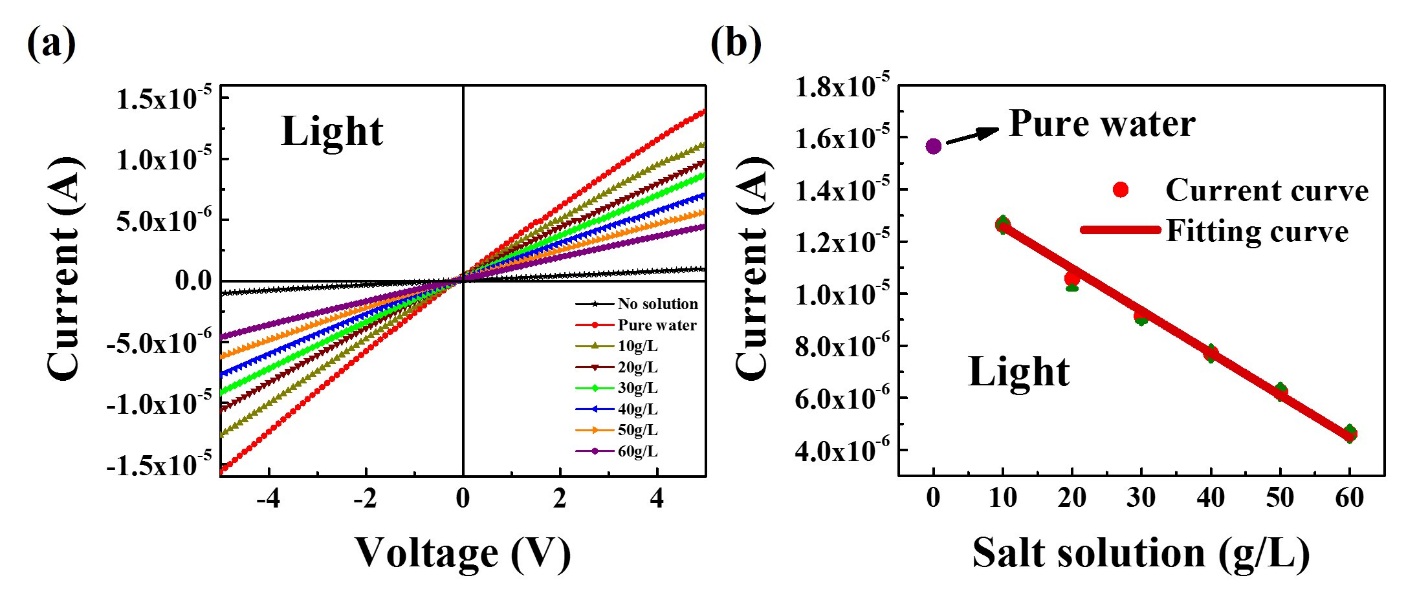


Figure S4 (a) The I-V characteristic curves of the sensor at different concentrations of salt solution under the light. (b) The photocurrent variation under different concentrations at the bias of 5V.

Sodium salts are dissolved in deionized water to make a series of salt solutions with concentrations of 10 g/L, 20 g/L, 30 g/L, 40 g/L, 50 g/L, and 60 g/L, respectively. Under the same conditions, the salt solution with these different concentrations is evenly sprayed on the surface of the chips and the current change of the sensor is shown in Figure S4. Figure S4 (a) shows the current of the sensor increasing sharply after spraying the deionized water. With increasing salt content in the solution the sensor current gradually decreases. At 5V bias, the variation in the device current as a function of solution concentration is shown in Figure S4(b), showing that the current decreases linearly with rising the concentration of salt solution.

The results in Figure S4 demonstrate that salt has an inhibitory effect on the hygroscopic of polyimide to suppress the current of the sensor.


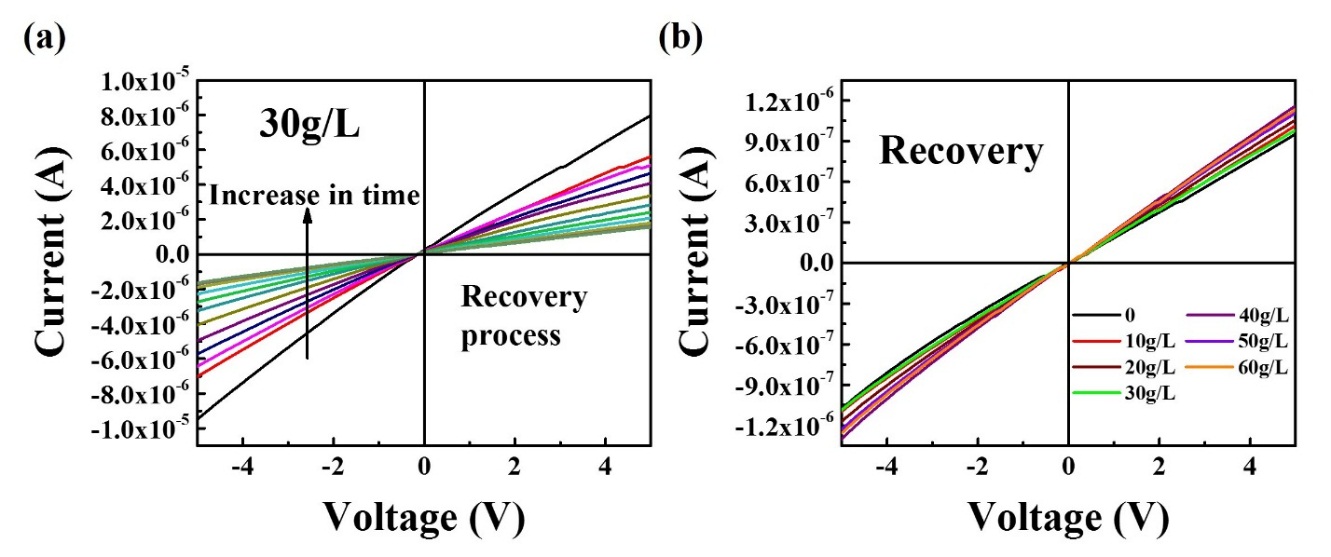


Figure S5 (a) The recovery process of the sensor after detecting salt solution at the concentration of 30g/L. (b) The recovery current of the different concentrations of salt solution.

After salt sensing test, the surface of the device is rinsed with deionized water to eliminate salt. The respective recovery process of the device performance over time is shown in Figure S5(a). With time going on, the sensor current diminishes to reach to the original state. Figure S5(b) shows the comparison of the recovery current and the original current of the device after testing at different concentrations of salt solution. When the water is completely volatilized, the device will eventually recover close to its original state.

In summary, our as-prepared sensors not only have excellent sensing performance, but also possess great reproducibility.


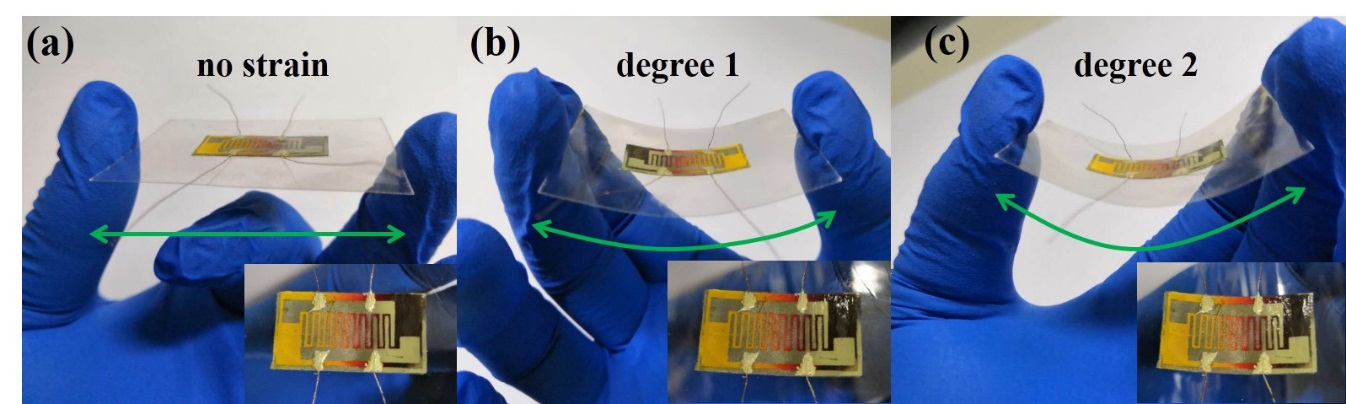


Figure S6. The wearable sensor with different bending degree.


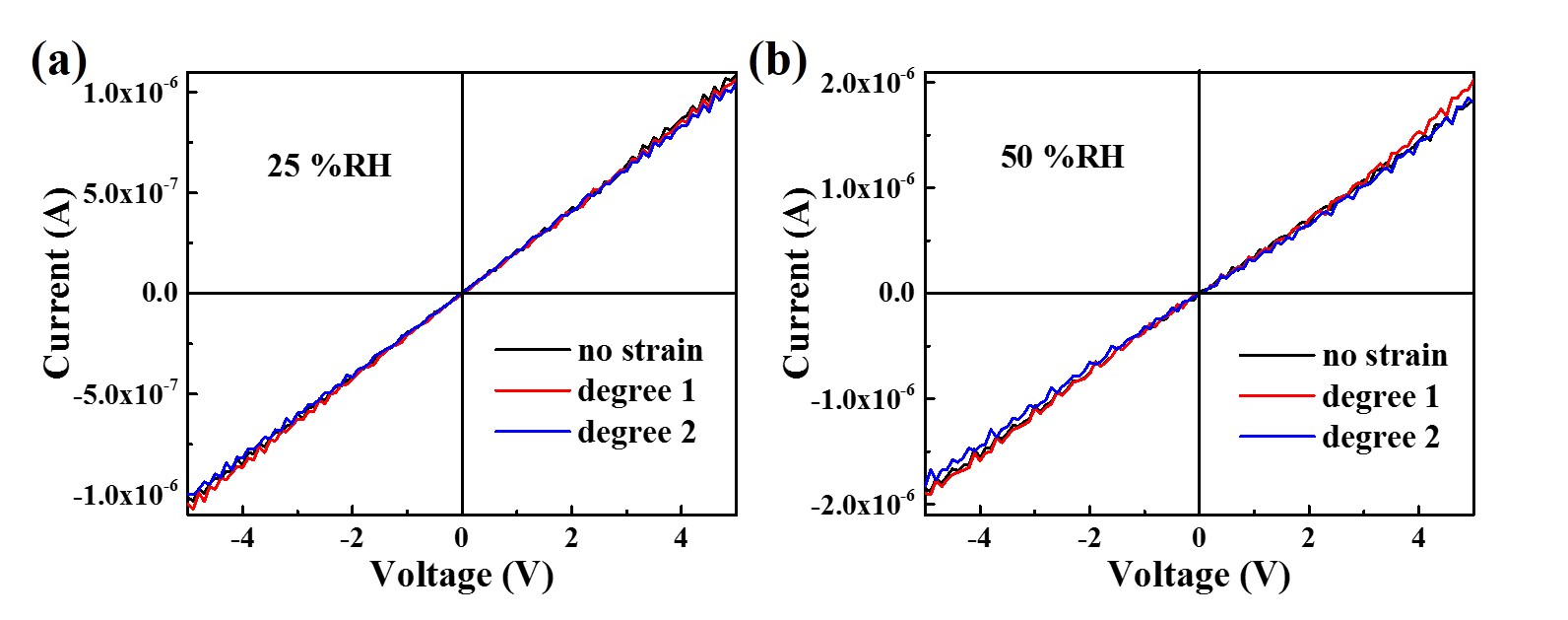


Figure S7. The I-V curves of the sensor with different bending degree at different relative humidity.

The fabricated wearable sensor has good mechanical properties and flexibility. As shown in Figure S6, the CdSSe and polyimide layer can still keep smooth and complete even at a large bending angle. The I-V curves at different bending conditions were exanimated in the same humidity (Figure S7). The drain-source current does not show any obvious difference, indicating the mechanical bending has almost no significant effect on the response characteristics of our sensors. Especially, in real-time sweat monitoring process, the bending angle is relatively small on body due to small area of the sensor. Therefore, the mechanical bending is no much influence on the performance of the wearable sensors.

As we known, almost all the reported wearable sweat sensors depend on the optical and chemical methods, for example, the ion concentration in sweat leads to the shift of the reflective peak or the ion concentration will change due to the electro-chemical reaction between the sensing materials and sweat, resulting in the variation of the current.

Our wearable sweat sensor originates a fresh new idea that is the dependence of expansion of polyimide on the moisture and salt volume makes stress on the CdSSe nanowire layers. Based on the variation of the photoconductivity, the sensitive detection of humidity, salt and sweat can be realized, to make the sensor can be fabricated with an easy way and has a good performance.

We have listed the comparison of performance of various reported wearable sensing materials in Table S1. It can be seen that our fabricated polyimide coated CdSSe wearable sensors show good responsibility of humidity, salt and sweat.

Table S1. Comparison of performance of various wearable sensing mast.

| Materials | Sensing | | Responsivity | Ref. |
| --- | --- | --- | --- | --- |
| Polyimide coated CdSSe nanowire chip | Humidity, salt and sweat | 244% | | This work |
| Oxide graphene (rGO)/inverse opal acetylcellulose (IOAC) | NaCl | 90% | | [1] |
| NH_2_-GO (Graphene Oxide)-Cu_3_(btc)_2_ | Sweat | 100% | | [2] |
| Au nanosheet, carbon nanotube, CoWO_4_, PDMS | Sweat | 70% | | [3] |
| Graphene oxide (rGO)-polyurethane | Humidity | 10% | | [4] |
| Polyvinyl alcohol (PVA)/KOH polymer gel electrolyte | Humidity | 30% | | [5] |

[1]. Xu H, Lu YF, Xiang JX, Zhang MK, Zhao YJ, Xie ZY, Gu ZZ. A multifunctional wearable sensor based on a graphene/inverse opal cellulose film for simultaneous, in situ monitoring of human motion and sweat. Nanoscale. 2018;10:2090-2098.

[2]. Wang Z, Gui M, Asif M, Yu Y, Dong S, Wang H, Wang W, Wang F, Xiao F, Liu H. A facile modular approach to the 2D oriented assembly MOF electrode for non-enzymatic sweat biosensors. Nanoscale. 2018;10:6629-6638.

[3]. Oh SY, Hong SY, Jeong YR, Yun J, Park H, Jin SW, Lee G, Oh JH, Lee H, Lee SS, Ha JS. Skin-Attachable, Stretchable Electrochemical Sweat Sensor for Glucose and pH Detection. ACS Appl Mater Interfaces. 2018;10:13729-13740.

[4]. Trung TQ, Duy LT, Ramasundaram S, Lee N-E. Transparent, stretchable, and rapid-response humidity sensor for body-attachable wearable electronics. Nano Research. 2017;10:2021-2033.

[5]. Li T, Li L, Sun H, Xu Y, Wang X, Luo H, Liu Z, Zhang T. Porous Ionic Membrane Based Flexible Humidity Sensor and its Multifunctional Applications. Adv Sci (Weinh). 2017;4:1600404.
